# Barriers and drivers to capacity-building in global mental health projects

**DOI:** 10.1186/s13033-020-00420-4

**Published:** 2020-12-03

**Authors:** Tarik Endale, Onaiza Qureshi, Grace Kathryn Ryan, Georgina Miguel Esponda, Ruth Verhey, Julian Eaton, Mary De Silva, Jill Murphy

**Affiliations:** 1 Department of Counseling and Clinical Psychology, Teachers College, Columbia University, 525 West 201th Street, New York, NY 10027 USA; 2 Department of Population Health, London School of Hygiene and Tropical Medicine, London, UK; 3 Health Service and Population Research Department, Institute of Psychiatry, Psychology and Neuroscience, King’s College London, London, UK; 4 ESRC Centre for Society and Mental Health, King’s College London, London, UK; 5 Research Support Centre, University of Zimbabwe, Harare, Zimbabwe; 6 Department of Population Health, London School of Hygiene and Tropical Medicine, London, UK; 7 CBM Global, Bensheim, Germany; 8 Department of Population Health, Wellcome Trust, London, UK; 9 Department of Psychiatry, Faculty of Medicine, University of British Columbia, Vancouver, BC Canada

**Keywords:** Global mental health, Capacity building, Implementation, Low- and middle-income countries, Training, Supervision, Quality assurance

## Abstract

**Background:**

The global shortage of mental health workers is a significant barrier to the implementation and scale-up of mental health services. Partially as a result of this shortage, approximately 85% of people with mental, neurological and substance-use disorders in low- and middle-income countries do not receive care. Consequently, developing and implementing scalable solutions for mental health capacity-building has been identified as a priority in global mental health. There remains limited evidence to inform best practices for capacity building in global mental health. As one in a series of four papers on factors affecting the implementation of mental health projects in low- and middle-income countries, this paper reflects on the experiences of global mental health grantees funded by Grand Challenges Canada, focusing on the barriers to and drivers of capacity-building.

**Methods:**

Between June 2014 and May 2017, current or former Grand Challenges Canada Global Mental Health grantees were recruited using purposive sampling. N = 29 grantees participated in semi-structured qualitative interviews, representing projects in Central America and the Caribbean (n = 4), South America (n = 1), West Africa (n = 4), East Africa (n = 6), South Asia (n = 11) and Southeast Asia (n = 3). Based on the results of a quantitative analysis of project outcomes using a portfolio-level Theory of Change framework, six key themes were identified as important to implementation success. As part of a larger multi-method study, this paper utilized a framework analysis to explore the themes related to capacity-building.

**Results:**

Study participants described barriers and facilitators to capacity building within three broad themes: (1) training, (2) supervision, and (3) quality assurance. Running throughout these thematic areas were the crosscutting themes of contextual understanding, human resources, and sustainability. Additionally, participants described approaches and mechanisms for successful capacity building.

**Conclusions:**

This study demonstrates the importance of capacity building to global mental health research and implementation, its relationship to stakeholder engagement and service delivery, and the implications for funders, implementers, and researchers alike. Investment in formative research, contextual understanding, stakeholder engagement, policy influence, and integration into existing systems of education and service delivery is crucial for the success of capacity building efforts.

## Background

The global shortage of mental health workers is a significant barrier to the implementation and scale-up of mental health services [1]. In low and middle income countries (LMICs) this shortage is estimated at 1.18 million mental health workers [2]. Partially as a result of human resource shortages, approximately 85% of people with mental, neurological and substance-use (MNS) disorders in LMICs do not receive care [3–5].

Task-sharing is an approach to addressing this “care gap” in LMICs, where mental health specialists are typically few and unevenly distributed [6]. Task-sharing is defined as shifting elements of the delivery of mental health interventions from mental health specialists to non-specialists [6]. There is mounting evidence of the effectiveness of task-sharing approaches for the management of MNS disorders [7]. Despite this, extremely few task-sharing interventions for mental health have been scaled up, and even fewer are sustained [8]. Consequently, developing and implementing scalable solutions for mental health capacity-building has been identified as a priority in global mental health (GMH) [9].

Evidence suggests that mental health training programs can improve provider behavior, intervention fidelity, and quality of service delivery [10]. Yet, globally, less than 2% of physicians and nurses have received training to recognize and treat patients with mental disorders in the last year [11]. Further, this training is often brief, with limited subsequent support, while findings of implementation research highlight the importance of ongoing supervision, coaching, and feedback [6]. In order to increase human resources for mental health, innovative and effective strategies for education, supervision and resourcing of mental health service providers, management of attrition, quality assurance, and leadership—not just training—are crucial [11]. Numerous studies cite capacity building as an integral component of GMH implementation in LMICs, with a variety of interventions and evaluation methods mentioned, suggesting a burgeoning knowledge base [12–14]. However, there remains limited evidence to inform best practices for capacity building in GMH [15].

As one in a series of four papers on factors affecting the implementation of GMH projects in LMICs, this paper reflects on the experiences of grantees funded by Grand Challenges Canada (GCC), exploring the barriers and drivers of capacity building.

## Methods

### Aims

This paper describes the qualitative analysis of barriers and drivers to capacity-building in GMH as part of a multi-method study examining implementation across a portfolio of GCC-funded projects (Miguel Esponda, et al. [16]). Based on the results of a quantitative analysis of GCC-funded project outcomes using a portfolio-level Theory of Change framework [17], six key themes were identified as important to implementation success: (1) Stakeholder engagement; (2) Training providers; (3) Supervision of providers; (4) Detection of mental illness; (5) Treatment of mental illness, and; (6) Prevention and promotion. This paper focuses on the themes related to training and supervision of providers, as well as quality assurance.

### Data collection

This study took place between June 2014 and May 2017. Current or former GCC Global Mental Health grantees were recruited using purposive sampling. Participants were approached during a GCC conference (2016) in London UK for interviews with members of the study team. Recruitment continued after the meeting with standardized participation templates, information sheets and consent forms sent by email. Interviews were conducted in-person or by Skype. N = 29 grantees participated in interviews, representing GCC-funded projects in Central America and the Caribbean (n = 4), South America (n = 1), West Africa (n = 4), East Africa (n = 6), South Asia (n = 11) and Southeast Asia (n = 3). Members of the research team conducted the interviews, which were recorded with the consent of participants. Interviews were 30–60 minutes and guided by a semi-structured interview guide which was developed to explore each step on a collective Theory of Change (ToC) map representing projects in GCC’s Global Mental Health funding portfolio, as described elsewhere in this volume (Miguel Esponda, et al. [16]). Grantees were asked to choose which steps they felt were the most important to discuss in relation to their projects, and to describe what helped or hindered their success in completing this step. Ethics approval was granted by the London School of Hygiene and Tropical Medicine’s Research Ethics Committee (#7746 and #9945).

**Fig. 1:**
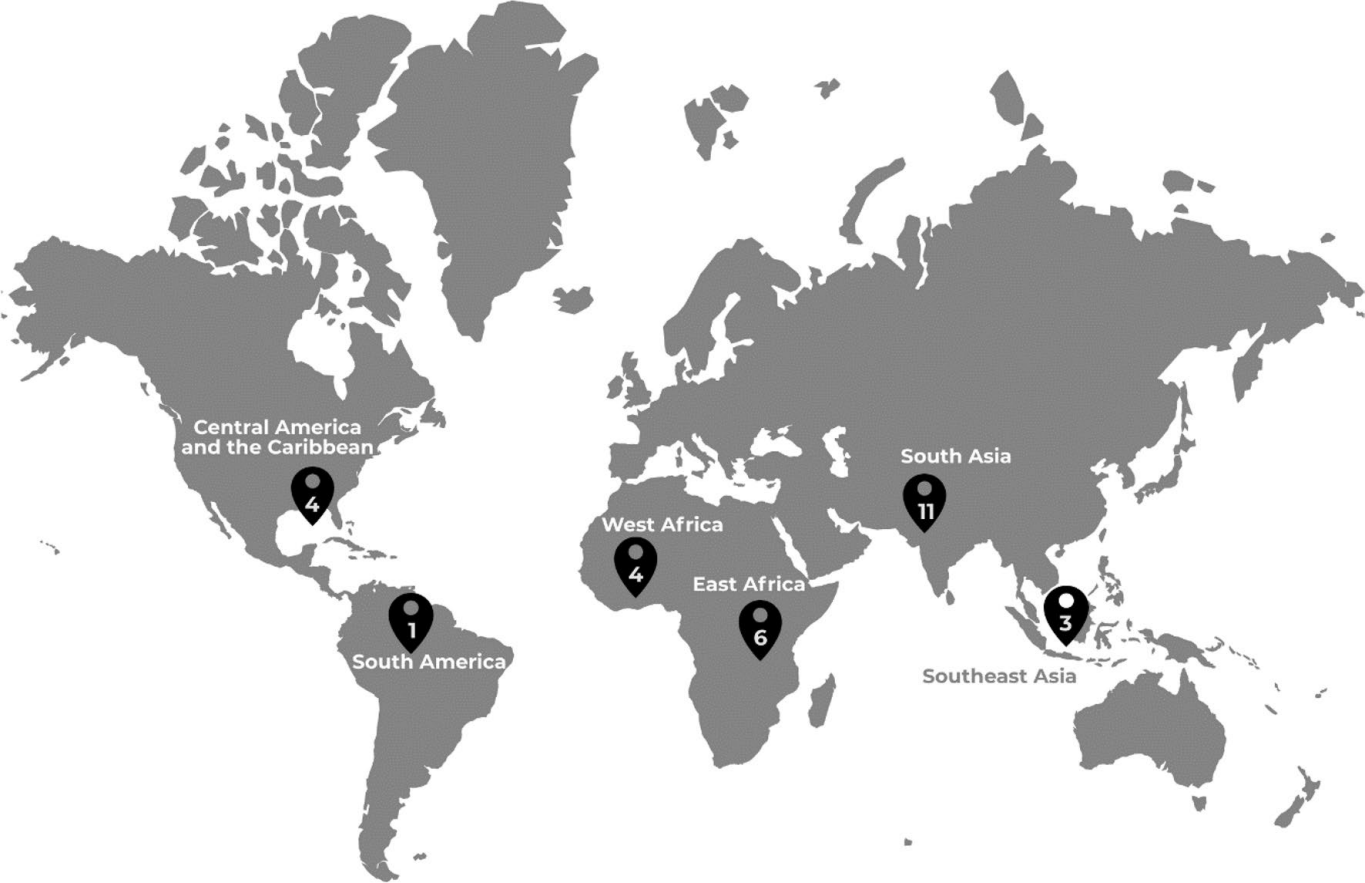
Geographic distribution of projects

### Data analysis

We applied a framework analysis approach, which has been widely used in health policy research to identify barriers and drivers [18]. Three members of the research team (JM, OQ, TE) coded transcripts using NVivo 11 software [19], with JM coding the full data set and OQ and TE each coding sixteen interviews. Following immersion in the data, JM developed an initial codebook, which was discussed and refined by the coding team. We coded three interviews using the refined codebook and ran a coding comparison in NVivo 11. The team then discussed areas of divergence, further refined the codebook, applied it to two additional interviews and developed a finalized version, which was applied to the remaining interviews.

**Fig. 2:**
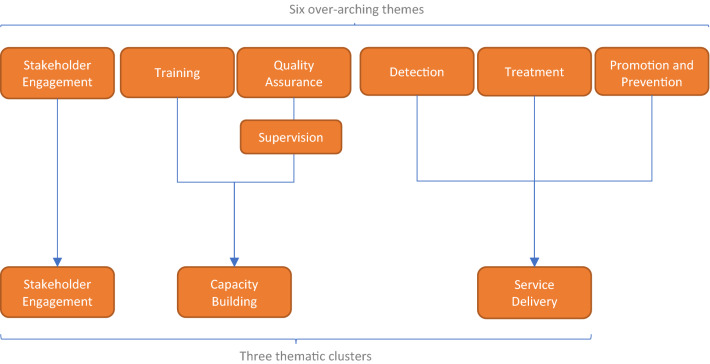
Breakdown of thematic clusters

Based on previous research and emerging results during analysis, the research team agreed that the six key themes should be grouped into three thematic clusters, as shown in Fig. 1: (1) Stakeholder Engagement (2) Capacity building, and (3) Service delivery (Fig. 2).

Following the initial coding process, we used the codebook to create an analytic framework which allowed for the identification of emerging themes for each of the three clusters. We populated the framework separately and then, following an iterative process, discussed and came to a consensus about predominant themes. This paper presents findings for the Capacity Building cluster, with findings from the other two clusters published elsewhere in this volume (Murphy, et al. [20]; Qureshi, et al. [21]).

## Results

Study participants described barriers and facilitators to capacity building within three broad themes: (1) training, (2) supervision, and (3) quality assurance. Responses regarding training, supervision, and quality assurance highlighted the importance of these activities as well as the challenges that arise during their implementation. Running throughout these thematic areas were the crosscutting themes of contextual understanding, human resources, and sustainability. Additionally, participants described approaches and mechanisms for successful capacity building which are summarised in Table 1.

**Table 1:** Approaches and mechanisms for successful capacity building

Theme	Approach/mechanism
Training	Training of Trainers approach and peer-to-peer learning promotes sustainability and adaptation to local context Refresher or booster trainings help maintain skills and promote fidelity Flexible training schedules or shorter sessions make it easier for busy providers to attend Low intensity training is easier to scale or pass on to local stakeholders Teaching problem solving skills (such as troubleshooting) builds independence Active learning approaches (group work, role playing, hands-on training) complement more theoretical or didactic approaches
Supervision	Regular face-to-face meetings or check-ins between staff and supervisors (when possible) foster strong relationships Taping sessions to be reviewed by a specialist can provide ongoing feedback and learning Periodic performance evaluation helps monitor increases in skill and knowledge and assess readiness for greater independence Debriefing with supervisors can help address challenges of service delivery
Quality Assurance	Client feedback can be used to monitor quality and satisfaction Audio-recorded sessions can be used to give feedback on quality of counselling sessions Effective mechanisms and approaches to supervision provides quality control (e.g. peer supervision) Using technology can support effective quality assurance processes

### Training

In relation to training, factors such as appropriateness and acceptability, retention, and time emerged as well as differences between training health sector and non-health sector providers. ‘Provider’ here is defined as mental health service providers, with ‘health sector’ referring to specialist or non-specialist health workers and ‘non-health sector’ referring to those in other sectors such as education, or lay workers such as peer counselors or traditional healers.

### Training of health sector providers-barriers

Many projects focus their training efforts on providers that function within formal health system settings (e.g. clinics or hospitals) but do not have specialized training in mental health (e.g. nurses or primary care doctors). Participants reported health system pressures and competing priorities as challenges to training these providers. In LMICs, high caseloads and multiple responsibilities are often spread across a limited workforce. This can make pulling health sector providers away from existing duties for initial training difficult. Afterward, integrating mental health training into practice within health systems can be challenging due to competing pressures and priorities:

“Training health workers – what does that mean? The health system[s] in low- and middle-income countries are not working, so every project starts with strengthening the system by at least training people. But is that the solution? I feel it is a short-term solution, because you have people trained, but no other supporting environment.” (Participant 19)

Contextual factors were reported as challenges to both receptivity to training and the ability to carry out trainings as planned. These ranged from gender norms influencing willingness to participate, to security issues and conflict preventing travel, to extreme weather events:

“So, the trouble was that as soon as we finished the first two days of training, there were the terrible floods... So, everything was impacted, some of the computers got destroyed… so what they did was they moved the whole operation of training higher up… and they basically did it out of make-shift places.” (Participant 25)

Differences in knowledge and beliefs also hindered training efforts. Stigma, unfamiliarity, and feelings of discomfort related to mental health and related topics such as human rights can present a challenge, especially when paired with the complexity of mental health issues that must be conveyed within the limited amount of time available for training health workers.

### Training of health sector providers-drivers

Two themes emerged relating to drivers for training of health sector providers. The first was the importance of motivation. Participants mentioned finding highly motivated people with an interest in mental health as an important contributor to the success of training. Many health workers were motivated by the offer of professional development opportunities and recognized the lack of mental health training otherwise available. The second theme was the importance of factors promoting the sustainability of training and the continuation of effects of training. Integration of mental health training for health sector providers into existing programs and curricula promotes this element of sustainability, as does selecting and training health workers who are connected to their communities:

“…one thing that might be positive in the long-run is the role of the [providers] becoming mental health workers, because they’re not going to go away once the intervention is done. They’re going to have the mental health training, and they’re going to remain in the community, and they’re going to make a connection to the people that they’re working with…” (Participant 12)

In addition to health sector providers’ motivation and connection to communities, attaining buy-in from policy makers was a key driver of sustainability and long-term success of training:

“We want primary care doctors to take ownership of this, so it’s very exciting for me that we’ve got to a stage where the directorate of health is saying, ‘Yes, please come and train them,’ so it’s the first step, and we are kind of hopeful that this will help to scale up one component of the program through the directorate of health services, without having to put in any extra funds.*”* (Participant 17)

### Training of non-health sector providers-challenges

The types of non-health sector providers mentioned by participants were diverse, and included schoolteachers, traditional healers, and general community members. This diversity sometimes presented difficulties, as skills and competencies among lay providers varied, and trainers sometimes found it challenging to tailor training to ensure consistent outcomes when baseline skills, competencies and experiences were so different.

Once training has been completed, there remains a challenge of maintaining quality and fidelity. Participants expressed concern regarding retention of skills as well as adherence to the intervention protocol among non-health sector providers, even when competency is ostensibly sound:

"We do pre- and post-training testing for competency. Of course… intervention enactment is another thing entirely. Yes, it’s not hard to recognize depression, they know how to treat, they are very good at it, so they are competent, fine. You clap for yourself, ‘Oh you’ve achieved so-and-so competency.’ But when it gets to doing the work, are you sure they are going to do what you want them to do? Even when you are not there? It’s another thing entirely." (Participant 21)

For some non-health sector providers, there are system pressures and priorities that differ from those of the health system, therefore presenting their own unique challenges. Bureaucratic structures and requirements in non-health sector public systems were burdensome and often caused delay of training:

"… you have to book things way in advance. Even for one of the [providers] to come to our training they had to have a piece of paper that came all the way from the [system administrators], all the way down to the [facility] and then to the [provider] itself [sic]. So, for every session that they attended they had to have all this paper work done.*”* (Participant 9)

### Training of non-health sector providers-drivers

Like with health sector providers, the benefit to sustainability that training local non-health sector providers offers was mentioned as a driver for success. Specifically, investing in capacity building at the local level was an important factor in the promotion of sustainability:

“What we want is actually to build their local capacity, so they can take it forward, because they are there to stay… So even if the project has stopped right now, they have those skills, they have those learnings, so they can continue working at least in their own community…” (Participant 22)

Fostering collaboration instead of competition between non-health sector providers and the formal medical system was also important for participation. This form of engagement provides an opportunity to tap into existing roles and capacities of providers such as traditional healers and religious or community leaders:

“…the good thing about [religious leaders] is that they’re going to do this anyway and this is what they’ve been doing, so there’s no need for additional resources really because we’re not asking them to become mental healthcare workers, we’re just asking them to add this [training] with whatever else they’re using in the way that they do it.” (Participant 29)

Capacity building of non-health sector providers at the local level also aids the scale up of mental health projects and the management of difficulties such as geographical challenges:

“Now if you are talking about scale, in one council there are about fifteen villages and you know there are rivers, there are mountains, the trains they are not connected, and everything like that, so there could be no one person roaming around and doing everything. So, you have to be innovative about it, and again, there were people who come from within the community, who said, ‘OK, you train us in supervision, we’ll do it, and we’ll roam around.’ Yeah. So, this is how adversity creates innovation!” (Participant 6)

Another driver of successful non-health sector provider training was the utilization of an iterative process. As a project progresses, ongoing learning can be used to hone training and selection processes over time, resulting in better training outcomes:

“But I think at the end, when we put people through the third round of training, the output was far better than the first two rounds… Initially we would just take anybody who was interested, right? Because we wanted to try it out. I think by the third time, we became better at selecting counselors who had the necessary skills to be good counselors.” (Participant 7)

### Training: appropriateness and acceptability-challenges

Participants noted the difficulties that cultural differences can present regarding appropriateness and acceptability. The varying acceptability of intervention specifics such as talking therapies or the shifting of traditional roles or views can challenge receptivity to training:

“There’s so much of these therapies that are really western in thinking, right? And they’re not, it’s not that they’re contradictory to the culture but they’re a little bit [different] than the ordinary thinking. That’s a shift, so it took our teachers a while to actually understand that, and I think we should give them the time to absorb this stuff, to repeat it.” (Participant 9)

Training and service delivery must also be perceived as meaningful or beneficial to providers to ensure uptake and follow through post-training:

“The health workers are going to be the ones who do this. You cannot force them, and you have to listen to them. Because you can’t just sit down here and have a ritual and think this is going to work… No. They just won’t do it. To them, it’s just one project, within a year you move on with your project, they stay, they’re still there in the health center. If you’re not going to change their life or make things easier for the health workers, they probably won’t support you.*”* (Participant 21)

### Training: appropriateness and acceptability-drivers

Regarding appropriateness and acceptability, participants also emphasized that adapting training to the local context was essential to success. This did not stop at translation, as adapting to the best manner of communication for the target audience meant more than just speaking in the same language as trainees:

“The critical thing that actually transformed it was when we got the first [leader] that we worked with, who was implementing the techniques in her school, to come and address them in their own language… we realized that different technical groups speak completely different languages, and if you are not careful you lose a lot in the translation… we had groups where even the senior educators don’t necessarily speak the language of the teachers.*”* (Participant 28)

### Training: retention-challenges

A common theme among participants was the difficulty of retaining a skilled workforce. A lack of mobility, career progression, and further opportunity in mental health contributed to movement towards better opportunities both within their country and outside of it. Participants emphasized that this was especially prevalent in settings with weak health, education, or economic systems. In some cases, capacity building itself threatened retention, as trainees became more eligible for outside opportunities:

“…people who are trained will always have a huge turnover rate because in those environments they make far less than they would make in a middle-income country or a high-income country. So there’s no incentives for them to stay…” (Participant 29)

Low rates of retention, and the additional trainings that were therefore necessary, resulted in larger numbers of trainees and further inputs required to overcome turnover– proving burdensome for project teams:

“The first training we did, I believe we trained 15-16 people but at the end we were only able to retain six, which meant that we then had to train double the amount of people that we needed. By the last training we trained three times as many people as we actually ended up with… and the amount of time and effort we put into capacity building and HR was a huge bulk of our time spent” (Participant 7)

### Training: retention-drivers

Alternatively, in some cases the projects provided meaningful work for well-educated people in contexts where there were low employment prospects or high rates of unemployment:

“…there were only two [lay health workers] that actually left the project before it was over. So the retention was not a problem. We said in our requirements for a lay health worker that they had to have high school education. Everyone who applied had at least some college… so it’s a reflection on more the lack of employment, especially in rural areas.” (Participant 22)

Providers finding meaning in the work—for example by seeing improvement in patients, or by training providers from (or with significant connections to) the target communities—were also drivers for success more generally:

“So then our group leaders are able to see the change in these women’s lives that they’ve facilitated so it’s a highly rewarding job. We have 23 facilitators today, next year we’re hiring another 25, we’ve never had a single group leader quit yet.*”* (Participant 15)

### Training: time-challenges

Challenges related to time served as barriers for both implementers and recipients of training. For implementers, capacity-building activities take time and delays cannot always be anticipated, often because of challenges mentioned earlier. This can lead to inconsistencies between reality and project goals or timelines, putting pressure on budgets:

“…we were supposed to train 320 primary health care workers… but the project was stopped for three months, and by the time it resumed, we had to retrain everybody all over again. So that, we spent so much money in trying to do that.” (Participant 21)

Similarly, the amount of time required to attend training sessions can be challenging for service providers due to both work schedules and other competing demands on time:

“Getting staff to be relieved from their regular work to attend those trainings is very difficult because most of these facilities work with very limited number of staff and so if you had to run a training… that can impact the service, so finding staff time has always been the most difficult part of doing the training.” (Participant 1)

These project timelines, service provider pressures, and challenging contextual factors mean that the limited available for training may not be sufficient to address more nuanced aspects of mental health—especially for health workers or other providers who have limited prior knowledge or training in mental health:

"…this is what is so different about mental health skills. Because these skills are internal skills as opposed to a drug or a surgical procedure… so it takes a little bit more time to absorb that, so that’s one issue, that we’re trying to find ways of actually shortening the learning curve...” (Participant 9)

### Training: time-drivers

The one driver for success mentioned by participants regarding time was shortening or breaking up training sessions, e.g. holding half-day sessions instead of full-day ones and integrating them into other training or routine activities. This made training providers with competing demands more feasible:

“This can be used in any kind of low or medium-income kind of setting, because the training is not too difficult… just two days of training for the maternal providers, they are able to do this a little bit more seamlessly within their routine provision of care.” (Participant 21)

### Supervision-challenges

Two major factors were identified as challenges for supervision. The first is the scarcity of human resources for management and supervision. The existence of these projects and their capacity building components is usually a result of the lack of human resources for mental health and often for health in general. This can make finding existing staff with the required skills for supervision difficult. Health providers may also be reluctant to take on the additional work and responsibilities attached to a supervisory role:

"…the whole idea was that we would train these medical officers to... to monitor and supervise the health workers… but they were not really interested or keen on getting an additional task being added to their worklist, because they’re generally seeing like 50–100 patients in a few hours’ time… But we’re doing all of this because we don’t have enough psychiatrists.” (Participant 17)

Logistical challenges often further complicate supervision models. Beneficiaries of these projects and the providers trained to reach them are often spread-out or in different locations than suitable supervisors. This can make common methods used to meet human resource challenges such as group supervision more difficult.

Participants also mentioned how resistant providers can be to supervision due to differing understandings of the practice or previous experiences with supervision. This can lead to the interpretation of supervision activities as critical or unhelpful rather than constructive.

“When you try and talk about supervision, people think you want to scrutinize them. Checking their things, checking on their weakness, checking on things they did not do. This is the way many people perceive, in the beginning, supervision… When they realize that what the supervisors were doing is not related to their first perception, to their beliefs, they realize that having someone supervising is more helpful than harmful.” (Participant 20)

### Supervision-drivers

The importance of investing in supervision and fostering strong relationships between supervisors and staff came up frequently. Respondents cited the utility of supervision for showing support and for monitoring the increase in skill and knowledge to ensure that providers are ready for greater independence. However, finding a balance between supervision and empowerment of providers was emphasized as it allows space for innovation and ownership. Shifting from specialist supervision to local or peer supervision was a commonly mentioned way to strike this balance, promoting both sustainability of capacity building efforts and empowering the recipients of training:

“...in each one of these programs, we’ve progressed from a supervisor-led supervision to eventually move it towards a peer-led supervision, because a supervisor, or an expert-led supervision is not sustainable. It’s not sustainable, it’s not scalable, we don’t have enough experts to do that.*”* (Participant 17)

### Quality assurance-challenges

Discussion of supervision frequently overlapped with the theme of quality assurance, as supervision can be integral to maintaining intervention fidelity and successful monitoring and evaluation, while quality assurance measures can feed back information to inform supervision. Suggested approaches to supervision often facilitated successful quality assurance, but there were distinct drivers and barriers as well. For example, participants described the challenge of measuring the complexity of their mental health project’s impact or outcomes. As one participant stated:

“It’s hard, because it’s a… multidimensional intervention, where we’re trying to impact social networks, we’re trying to impact self-esteem, we’re trying to impact violence issues, communication, so it’s a lot of different dimensions. And then of course, psychosocial, health, well-being, knowledge, all of these things!” (Participant 16)

Contextual elements, such as variations in language or informal delivery settings, and unexpected events such as natural disasters, can challenge the validity and reliability of quality assurance methods or confound the outcomes of interventions:

“They may have improved in terms of the scale of depression, but then this trauma happens, so they may rightly be more depressed than when we actually got them… it may seem as if there was no improvement, but there may have been [an] improvement but that was lost due to the hurricane.*” *(Participant 29)

Quality assurance processes and logistics can be resource intensive, especially when implemented in low-resource settings that require investment in infrastructure or technology, such as telephone lines to allow communication, or when adapting to the above-mentioned contextual challenges. This can hinder quality assurance capacity by limiting the scope, regularity, or method of assessment. A tension between being flexible enough to allow situational adaptation during implementation and retaining fidelity in the name of quality assurance was described:

“…one of the things that we’ve found is that people change the delivery methods because they know how to best apply it to their particular situation. And when we were working with the four schools, there were differences throughout the entire four schools. The process, however, was completely the same. The differences were in the delivery… but the overall change in behavior was the same, in other words the children still improved.” (Participant 28)

Participants also highlighted the need for benchmarks to help interpret the findings of quality assurance and monitoring and evaluation (M&E) assessments and inform implementation and decision-making:

“And even if you’re at thirty percent, is that bad or good? On one hand it’s bad but on the other hand that’s thirty percent who are now depression-free who without these groups would certainly have been depressed. So, we have to decide at what level do we want to reinvest our resources to improve that quality… when do we call in the cavalry to get the quality back up? What’s our minimum threshold before the red light goes off and the production line stops and we have to fix it?*”* (Participant 15)

### Quality assurance-drivers

The use of effective frameworks to help capture pathways and mechanisms of implementation was considered a key driver for successful quality assurance. As a participant explained about using one such methodology (which was required by GCC), Theory of Change:

“But when you implement variables that you don’t know about those are the ones that determine that – variables such as the fact that teachers don’t like doctors. That’s a real thing, something like that can throw off everything. And so it’s a whole different kettle of fish, so that’s why I think the Theory of Change is one of the critical things for successful implementation, after you have a product that has been proven to be effective. Because developing efficacy is the first step, it doesn’t translate to success.” (Participant 28) 

Continued monitoring and evaluation beyond the timeframe of initial implementation, especially if service delivery is integrated into and handed off to the government health system, was also seen as essential for successful scale-up.

### Approaches and mechanisms

Finally, participants shared various approaches and mechanisms they found important to successful capacity building for mental health projects, which are summarized in the Table 1.

## Discussion

Capacity building, namely through the themes of training, supervision, and quality assurance, came up frequently in respondent interviews, demonstrating its importance to GMH implementation and its place among related barriers and drivers to success. Though capacity building has been previously identified as a crucial component of GMH implementation and important strategies for some components such as training have been flagged, capacity building activities are rarely described with any detail [15] (with notable exceptions [6, 13, 14]). The results of this study point to several strategies that can help overcome barriers and tap into drivers for successful GMH implementation (Table 2).

**Table 2:** Themes and recommendations

Theme	Recommendations
Contextual Understanding	Invest in formative research to better understand context
	Look for opportunities to “build back better” after emergencies. Recognise and capitalise on ‘policy windows’ in a timely manner
Human resources	Carefully identify appropriate cadres and individuals to train
	Provide meaningful and beneficial training, relationships, and opportunities to promote motivation and retention
Sustainability	Integrate training into existing health and education systems
	Invest in specialist training, reliable medication supplies, and referral networks to support task-sharing initiatives and trained personnel
	Invest in buy-in from service providers, educators, and policy makers
Quality assurance	Research the tension between appropriate and sustainable approaches and evidence based practices
	Develop and use standards to guide evaluation, interpretation of results and subsequent decision making
	Use theoretical frameworks such as Theory of Change and participatory approaches

The importance of understanding and accounting for contextual factors emerged throughout the results. From mapping how service providers fit into larger systems, to understanding how cultural factors may interact with training, supervision, and quality assurance processes, contextual understanding–or a lack thereof–can aid or severely impede implementation efforts [22]. This fits with previous research findings; for example, the ‘Emerging mental health systems in low- and middle-income countries’ (Emerald) program found that despite similarities across six countries, capacity building had to be tailored to meet the specific context of each setting [13]. Similarly, participants cited factors that cannot always be anticipated through formative research, such as natural disasters, as barriers to successful implementation. Understandably, such events can be incredibly disruptive. However, multiple case studies [23, 24] and WHO recommendations have highlighted the important “policy window” that can arise from these emergencies, by tapping into the influx of attention and resources to “build back better” [25].

Predictably, human resources featured as a central component of participant responses, as the lack of mental health workers is a consistent challenge and provides the impetus for capacity building efforts [1, 2, 5]. However, this emerging theme of human resources goes beyond the often-limited focus on training itself, and has implications for factors such as fidelity and sustainability. For example, retention of mental health providers, both during and after training, frequently presents as a barrier in this study’s findings and in other literature [22]. Motivation, in terms of understanding what drives service providers and keeping their motivation high, serves as an important point within this theme of human resources. Providing training, professional relationships, and opportunities that providers see as meaningful, beneficial to themselves and their patients, and useful for professional development, can promote effective training, retention, and sustainability [6, 22].

Sustainability is a common topic in global health and one that ran through every major theme of this study. Participants highlighted concerns about what long-term impact training has if nothing is integrated into or improved within surrounding systems of health and education and no feeling of ownership is cultivated beyond the implementation team. This resonates with previous findings highlighting the importance of simultaneously strengthening specialist services as task-sharing occurs, improving supervision, training, and referral networks, developing reliable supplies of psychotropic medication, and supporting the provision of psychosocial interventions to augment medicine-based approaches in primary care [26]. In this regard, stakeholder engagement (Murphy, et al. [20]) becomes extremely important to the sustainability of capacity building efforts as buy-in from service providers, educators, and policy-makers impacts success long before training begins and well after the last training session is completed.

The importance (and difficulty) of quality assurance appeared in different forms. There was a tension between the flexibility required to create the most appropriate and sustainable approaches and empower service providers and the desire to follow the evidence base and ensure fidelity and strong supervision. Quality assurance processes, such as monitoring, evaluation, and supervision, can be incredibly time and resource intensive and are susceptible to contextual challenges such as geography and infrastructure [27]. Programs described in the GMH literature are often characterized by manualized interventions that lack guidelines for evaluating competency, fidelity, and quality [28]. Participants also described the difficulties of evaluating complex interventions while considering potential confounding variables in their given contexts. Even when these processes are successfully navigated, understanding the implications of the results can be difficult without a set of standards to guide further decision-making [29]. Using theoretical frameworks such as a Theory of Change [17] and tools to assess competencies [28, 30, 31] can help map out the complexity, encourage a participatory approach, and guide evaluation and future decision making for mental health capacity building [32].

Many of the approaches and mechanisms recommended by participants for effective training, supervision, and quality assurance (see Table 1) mirror the literature on capacity building [6]. However, much of this information comes from implementation research from high-income countries, meaning this study builds on growing evidence that many of these approaches are also applicable in LMICs [6, 15]. A noticeable area of interest that did not feature in this study was the importance of capacity building to promote the involvement of service users and to strengthen mental health system governance. Training for service users, caregivers, and service providers to promote inclusion can help improve the appropriateness and quality of services and offer greater protection for service users [13, 33, 34], and poor governance has been identified as a key barrier to effective mental health implementation [12, 13, 35]. However, this omission may be a reflection of a wider absence of information [36, 37] despite the recognition of this type of capacity building’s importance [2].

### Limitations

This study sought to capture the experiences of GCC-funded grantees, meaning that the viewpoints of those targeted by capacity building efforts and in many cases those providing the direct training and supervision were not captured. Additionally, interviews were only conducted in English, potentially omitting responses from non-English speaking grantees. A diverse pool of grantees responded, including from non-Anglophone countries, but these and other factors further detailed by Murphy, et al. ([20]) might have limited the gathering of an even more valuable perspectives.

## Conclusions

This study identified drivers and challenges to capacity building during the implementation of global mental health projects. It also demonstrates the importance of capacity building to GMH research and implementation, its relationship to stakeholder engagement and service delivery, and the implications for funders, implementers, and researchers alike. Investment of time and money into formative research, stakeholder engagement, policy influence, and integration into existing systems of education and service delivery is crucial for the success of capacity building efforts. Without in-depth contextual understanding, interventions are doomed to irrelevance. Without stakeholder engagement, there will be no buy-in for the development or success of an intervention. And without this buy-in, integration into existing systems of education and service delivery are impossible, leaving projects with no funding or policy support to sustain them. Attention to, and investment in, GMH is increasing rapidly, and without substantial investment in local capacity development, there is a risk of poor implementation of projects. Capacity building, which will need to be carried out in advance of substantial investment at country level, is therefore a core component of ensuring effective and impactful future scale-up of projects to close the care gap in mental health. Careful attention to learning from previous experiences is crucial to ensuring high quality efforts to improve both implementation and absorption capacity for future funding.

## Data Availability

The quantitative data that support the findings of this study are available from GCC, but restrictions apply to the availability of these data, which were used under license for the current study, and so are not publicly available. These data are however available from the authors upon reasonable request and with permission of GCC.

## Abbreviations

LMICs: Low- and middle-income countries
MNS Disorders: Mental, neurological, and substance use disorders
GMH: Global mental health
GCC: Grand Challenges Canada
WHO: World Health Organization
